# Potent Antiproliferative Effect on Liver Cancer of Medicinal Plants Selected from the Thai/Lanna Medicinal Plant Recipe Database “MANOSROI III”

**DOI:** 10.1155/2015/397181

**Published:** 2015-06-07

**Authors:** Aranya Manosroi, Hiroyuki Akazawa, Worapong Kitdamrongtham, Toshihiro Akihisa, Worapaka Manosroi, Jiradej Manosroi

**Affiliations:** ^1^Faculty of Pharmacy, Chiang Mai University, Chiang Mai 50200, Thailand; ^2^Manose Health and Beauty Research Center, Chiang Mai 50200, Thailand; ^3^Department of Biotechnology and Material Chemistry, Nihon University Junior College, Chiba 274-8501, Japan; ^4^College of Science and Technology, Nihon University, Tokyo 101-8308, Japan; ^5^Akihisa Medical Clinic, 1086-3 Kamo, Sanda-shi, Hyogo 669-1331, Japan; ^6^Faculty of Medicine, Chiang Mai University, Chiang Mai 50200, Thailand

## Abstract

Thai/Lanna medicinal plant recipes have been used for the treatment of several diseases including liver cancer. In this study, methanolic extracts (MEs) of 23 plants were tested for antiproliferative activity on human hepatoma cell line (Hep G2) by the sulforhodamine B (SRB) assay. Nine MEs with potent antiproliferative activity (IC_50_ < 100 *µ*g/mL) were obtained and further semipurified by liquid/liquid partition extraction. The semipurified fractions were tested for the antiproliferative and antioxidative activities. ME of *Stemona collinsae* and the semipurified extract and methanol-water fraction (MF) of *Gloriosa superba* gave the highest antiproliferative activity on HepG2 which were 4.79- and 50.07-fold cisplatin, respectively. The semipurified fractions showed an increased antiproliferative activity. MF of *Caesalpinia sappan* and HF of *Senna alata* showed the highest free radical scavenging and metal chelating activities, respectively. The compound in *n*-hexane fraction (HF) of *Ventilago denticulata* which showed an increase in antiproliferative activity comparing to its ME was isolated and identified as emodin. This study has demonstrated the potential of the ME from *S. collinsae*, MF from *G. superba*, and emodin isolated from *V. denticulata*, for further development as an antiliver cancer agent.

## 1. Introduction

Hepatocellular cancer and intrahepatic cholangiocarcinoma are the two major forms of primary liver cancers. The incidence of these two carcinomas is the fifth most common widespread cancer in the world. Hepatocellular carcinoma can damage the hepatic cells via oxidative stress and generation of inflammation related to hepatocarcinogenesis [1]. Several types of chemotherapeutic drugs have been used to treat hepatic cancer such as cisplatin, 5-fluorouracil, and paclitaxel. However, these drugs usually have some problems of cancer resistance, due to multidrug resistance protein and the decrease of apoptotic proteins. Recently, many medicinal plants have been investigated as an alternative of chemotherapeutic drugs. Most medicinal plants from traditional medicines are safer than the chemical substances with effectiveness. This may be due to the traditional uses in human for several generations. Only those which showed therapeutic efficacy and safety will be recorded and used until now.

The Lanna region covered several provinces in China, Laos, Myanmar, and Thailand. The Lanna region in Thailand includes seven provinces which are Chiang Mai, Chiang Rai, Lamphun, Lampang, Phayao, Phrae, and Nan. Lanna has its own folklore wisdom including traditional medicinal plant recipes. These recipes have been recorded and used for over 700 years. Interestingly, some of the recipes are still currently used in the northern part of Thailand for the treatment of several diseases such as fever, diarrhea, diabetes, tuberculosis, arthritis, and cancer. Professor Dr. Jiradej Manosroi from the Manose Research Center has collected the medicinal plant recipes from several institutions, temples, and folklore doctors in the Lanna area and other regions all over Thailand and put into the database called “MANOSROI III.” Presently, this database contains about 200,000 medicinal plant recipes covering all diseases including cosmetics and food supplements [2, 3].

Anticancer activities of many Thai medicinal plants can be selected from the recipes in the “MANOSROI III” database. Some plants have shown potent antiproliferative activities* via* apoptotic action [3]. In this study, the antiproliferative activity on human hepatoma cell line (HepG2) and antioxidative activities of the methanol extracts of 23 plants selected from the Thai medicinal recipes database “MANOSROI III” were investigated. The extracts with strong antiproliferative activity were selected, further purified by liquid-liquid partition extraction, and reinvestigated and the potential to develop as anticancer compounds was anticipated.

## 2. Materials and Methods

### 2.1. Materials and Chemicals

Twenty-three plants with high frequency in the anticancer recipes were selected from the anticancer recipes in the Thai/Lanna medicinal recipe database “MANOSROI III.” Searching of the anticancer recipes was performed by using Thai keywords which were Ma-reng and San which mean cancer. The plants were collected from Chiang Mai, Thailand during March to May 2010. The voucher specimens of the plants were authenticated by a botanist and deposited at NPRDC, Faculty of Pharmacy, Chiang Mai University in Chiang Mai, Thailand (Table 1). Methanol was obtained from Labscan Asia Co. Ltd. (Thailand). Fetal bovine serum (FBS) was from Gibco BRL (Ontario, Canada). Dulbecco's Modified Eagle Medium (DMEM) cell culture medium, dimethylsulfoxide (DMSO), sulforhodamine B (SRB), l-(+)-ascorbic acid, *α*-tocopherol, 1,1-diphenyl-2-picrylhydrazyl (DPPH), ethylenediaminetetraacetic acid (EDTA), ferrozine, ferrous chloride (FeCl_2_), Folin-Ciocalteu reagent, aluminium chloride (AlCl_3_), potassium acetate (CH_3_CO_2_K), and quercetin were purchased from Sigma Chemical Co. (St. Louis, MO, USA). Cisplatin and doxorubicin hydrochloride (doxorubicin) were from Dabur Pharma Ltd. (Hampshire, UK).

### 2.2. Crude Plant Extract Preparation

Ten grams of the dried and powdered plants was extracted with 200 mL of methanol (2 h reflux, 3 times). The mixture was filtered and the methanol filtrate was evaporated under reduced pressure using a rotary evaporator (Buchi, Switzerland) to give the methanol extract (ME). The yield was represented as percentages of the crude extracts comparing to the dried plant.

### 2.3. Liquid-Liquid Partition Extraction

**Figure 1:**
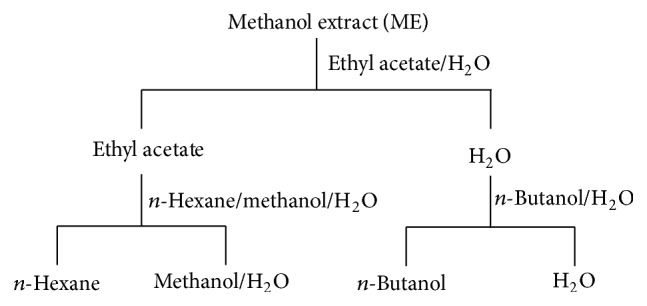
Liquid-liquid partition method from the methanolic extract (ME) to the 4 fractions (*n*-hexane, methanol-water, *n*-butanol, and water soluble fractions).

The 9 MEs which gave the GI_50_ values of less than 100 *μ*g/mL [active (A)/moderately active (MA) according to the NCI criteria] were selected and further fractionated by liquid-liquid partition extraction. Briefly, one gram of the MEs was dissolved in 100 mL of water and partitioned with an equal volume of ethyl acetate. The aqueous layer was reextracted with an equal volume of ethyl acetate for 2 times. The ethyl acetate layer was pooled, concentrated to 5 mL, dissolved in 95 mL of methanol, and reextracted with 100 mL of hexane for 3 times to obtain the hexane (HF) and methanol-water (MF) soluble fractions. The aqueous layer was reextracted with an equal volume of *n*-butanol for 3 times to obtain *n*-butanol (BF) and water (WF) soluble fractions (Figure 1).

### 2.4. Compound Isolation from* Ventilago denticulata*

The dried stem of* V. denticulata* (1.6 kg) was extracted by methanol to obtain ME (100 g). A portion of ME (78 g) was extracted by liquid-liquid partition extraction to yield HF (15 g), MF (14 g), BF (17 g), and WF (16 g). According to the antiproliferative activity results, HF (2.6 g) was further fractionated by silica gel column chromatography (50 g) using the mixture of hexane and ethyl acetate as the mobile phase. Seven fractions (HF-1~7) according to the successive polarity range solvents (hexane/ethyl acetate = 1 : 0 to 0 : 1) were obtained. Fraction HF-2 (1.3 g) which was eluted by 90% hexane in ethyl acetate was further purified by silica gel column chromatography (50 g) using hexane-ethyl acetate to obtain the orange fraction (HF-2-O, hexane/ethyl acetate = 95 : 5). This fraction was purified by the preparative HPLC with C18 column (10*ϕ* × 250 mm, 5 *μ*) using methanol/water = 9 : 1 and the flow rate at 2.0 mL/min to obtain a single compound.

### 2.5. Antiproliferative Activity

#### 2.5.1. Sample Preparation

The MEs and fractions were dissolved in DMEM medium containing 0.5% v/v of DMSO [4] and DMSO, respectively. The samples were centrifuged at 5,000 g, room temperature (25 ± 2°C) for 5 min, sterilized by filtering through 0.2 *μ*m cellulose acetate membranes (Sartorius, Göttingen, Germany), and stored at −20°C. The final concentrations of MEs and fractions were in the range of 1 × 10^1^–1 × 10^−5^ and 1–1 × 10^−6^ mg/mL, respectively.

#### 2.5.2. Human Hepatic Cancer Cell Culture

The human hepatoma (Hep G2) cell line was obtained from Faculty of Tropical Medicine, Mahidol University, Bangkok, Thailand. The cells were subcultured into a 25 cm^2^ plastic flask containing DMEM supplemented with 10% of FBS, 100 U/mL of penicillin, and 100 mg/mL of streptomycin. The flask was incubated at 37°C in a humidified air incubator containing 5% carbon dioxide (CO_2_).

#### 2.5.3. Antiproliferative Activity by the Sulforhodamine B (SRB) Assay

Effects of extracts on the growth of Hep G2 cell line were evaluated according to the procedure of the American National Cancer Institute (NCI) for the* in vitro* anticancer drug screening using the protein-binding dye SRB to assess cell growth [5]. The assay was performed as previously described by Manosroi et al. [6] with some modifications. Briefly, cells were harvested, seeded into 96-well plate (Gibthai Co. Ltd. Thailand) at the density of 1 × 10^4^ cells/well, and incubated for 24 h at 37°C in a 5% CO_2_ incubator. The cells were treated with various concentrations (10–10^−6 ^mg/mL) of the samples for 24 hours. After incubation, the cells were fixed with 50% trichloroacetic acid and dyed with SRB solution. One hundred *μ*L of Tris-solution was added to each well and incubated for 30 min. The absorbance was measured at 540 nm by a microplate reader (Bio-Rad, model 680, Philadelphia, PA, USA). The final concentration of DMSO in each well was less than 1% v/v and the assays were done in triplicate. The percentage of growth inhibition (%*G*) was calculated as %*G* = (*T*_treat_ − *T*_0_)/(*T*_control_ − *T*_0_) × 100, where *T*_treat_ was the absorbance of the samples after incubation for 24 h, *T*_0_ was the absorbance at initial (0 h), and *T*_control_ was the absorbance of the control (DMSO only) after incubation for 24 h. The dose response curve was prepared by plotting the %*G* versus the concentrations of the samples and the IC_50_ values (the concentrations of the samples giving 50% growth inhibition) were determined. Cisplatin and doxorubicin, the standard anticancer drugs, were used as the positive controls. Folds of activity in comparing with the standard drugs were calculated as follows: Folds = (IC_50_ values of the standard drugs)/(IC_50_ values of the samples).

### 2.6. Antioxidative Activities

#### 2.6.1. 1,1-Diphenyl-2-Picrylhydrazyl (DPPH) Radical Scavenging Activity

The DPPH radical scavenging activity was determined by the method previously described [7]. Briefly, 50 *μ*L of the samples was put into 96-well microplate (Nalge Nunc International, NY, USA) and 50 *μ*L of ethanol solution of DPPH were added into each well and mixed. After incubation for 30 min at room temperature (25 ± 2°C), the absorbance was measured at 515 nm by a well plate reader (Bio-Rad, model 680 microplate reader, PA, USA) against the blank (DMSO). All samples were dissolved in DMSO and the final concentrations were adjusted to the range of 0.01–100 *μ*g/mL. Ascorbic acid and *α*-tocopherol were used as the positive controls. The experiments were done in triplicate. The SC_50_ value, the concentration of the sample that scavenged 50% of DPPH radical, was determined from the plotted curve.

#### 2.6.2. Metal Chelating Activity

The metal chelating activity was determined by the ferrous iron-ferrozine complex method as previously described [7]. The mixture of the sample (50 *μ*L) and water (20 *μ*L) was added into a 96-well plate and 1.5 mM FeCl_2_ (10 *μ*L) in water was added into each well and mixed. After incubation for 15 min at room temperature (25 ± 2°C) in darkness, 20 *μ*L of 3 mM ferrozine in water was added into each well and the absorbance was measured by a microplate reader at 570 nm using DMSO as a negative control. The final concentration of the samples was varied within the range of 0.005–500 *μ*g/mL. EDTA was used as a positive control and the experiments were done in triplicate. The concentration in which the sample exhibited 50% metal chelating activity of ferrous ion (CC_50_) was determined.

#### 2.6.3. Total Phenolic Contents

The total phenolic contents as quercetin equivalents of the extracts and fractions were determined using the Folin-Ciocalteu reagent [7]. Briefly, 10 mg/mL of the samples was mixed with Folin-Ciocalteu reagent and 20% (w/v) of sodium carbonate (Na_2_CO_3_) at room temperature (25 ± 2°C). After incubation for 60 min, the absorbance at 760 nm was measured by a microplate reader. The total phenolic contents of the samples were expressed as *μ*g of quercetin equivalents (QE) per mg of the samples.

#### 2.6.4. Total Flavonoid Contents

Aluminium chloride (AlCl_3_) colorimetric method was used to determine the flavonoid contents with slight modification [8]. Briefly, 10 mg/mL of the samples was mixed with methanol, 5 *μ*L of 10% AlCl_3_, 5 *μ*L of 1 M CH_3_CO_2_K, and 140 *μ*L of distilled water and the mixture was adjusted to 250 *μ*L with methanol. After incubation at room temperature (25 ± 2°C) for 30 min, the absorbance at 415 nm was measured with a microplate reader. The total flavonoid contents of the samples were expressed as *μ*g of quercetin equivalents (QE) per mg of the samples.

### 2.7. Statistical Analysis

All assays were performed in triplicate of three independent and separate experiments. The means of each test were calculated.

## 3. Results

### 3.1. Antiproliferative Activity of the 23 Methanolic Plant Extracts

Botanical names, families, parts used, yields, and antiproliferative activity on Hep G2 cell line of the 23 Thai medicinal plant MEs were shown in Table 1. According to the cytotoxicity activity criteria as established by the NCI, the IC_50_ values lower than 20 *μ*g/mL, in the range of 20 to 100 *μ*g/mL, and more than 100 *μ*g/mL are regarded as active (A), moderately active (MA), and inactive (IA), respectively [9]. Only ME of* S. collinsae* showed A activity with the highest antiproliferative activities (IC_50_ = 5.51 *μ*g/mL) on HepG2 cell line which was 4.79- and 0.10-fold cisplatin and doxorubicin, respectively. Eight MEs of* G. superba* (IC_50_ = 20.11 *μ*g/mL),* C. sappan* (IC_50_ = 20.12 *μ*g/mL),* V. denticulata* (IC_50_ = 20.88 *μ*g/mL),* S. alata* (IC_50_ = 22.46 *μ*g/mL),* Rhinacanthus nasutus* (IC_50_ = 30.91 *μ*g/mL),* Albizia chinensis* (IC_50_ = 34.50 *μ*g/mL),* Pouzolzia pentandra* (IC_50_ = 35.51 *μ*g/mL), and* Fibraurea tinctoria* (IC_50_ = 89.11 *μ*g/mL) which gave the IC_50_ values of less than 100 *μ*g/mL were regarded as MA. The A and MA MEs and their fractions were further fractionated, tested for antiproliferative activity of these 9 MEs, and summarized in Table 2. The* G. superba* MF extract exhibited the highest antiproliferative activity with the IC_50_ value of 0.53 *μ*g/mL (A) which was 37.79-, 50.07-, and 1.02-fold its ME, cisplatin, and doxorubicin, respectively.

### 3.2. Antioxidative Activities of 9 MEs and Their Fractions

The antioxidative activities of 9 MEs and their fractions were presented in Table 2. The MF of* C. sappan* exhibited the highest DPPH radical scavenging activity with the SC_50_ of 21.91 *μ*g/mL which was 1.09- and 0.84-fold *α*-tocopherol (SC_50_ = 23.80 *μ*g/mL) and ascorbic acid (SC_50_ = 18.30 *μ*g/mL), respectively. HF of* S*.* alata* gave the highest metal chelating activity with the CC_50_ of 45.60 *μ*g/mL which was 0.28-fold EDTA, the standard chelating agent (CC_50_ = 12.90 *μ*g/mL). Interestingly, the high phenolic and flavonoid contents were related to the strong antioxidative DPPH radical scavenging and metal chelating activities (Table 2). ME of* C*.* sappan* showed the highest phenolic contents with the QE of 475.41 *μ*g/mg, whereas the MF of* R*.* nasutus* gave the highest flavonoid contents with the QE of 35.91 *μ*g/mg.

### 3.3. Antiproliferative and Oxidative Activities of the Semipurified Fractions from HF of* V. denticulata*

**Figure 2:**
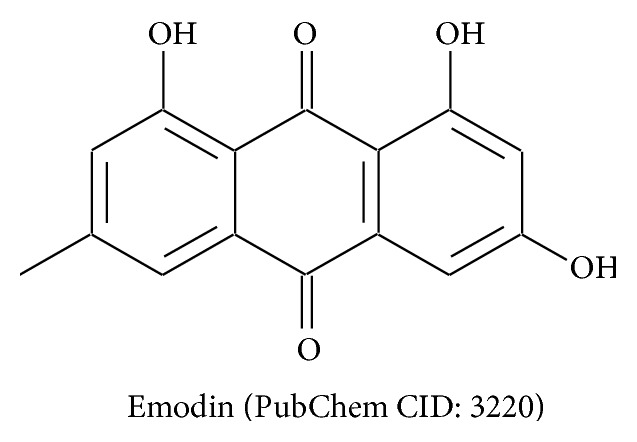
The structure of isolated compound from the HF-2 fraction of* V*.* denticulata* stem.

Only HF of* V*.* denticulata* was further semipurified by column chromatography using polarity range solvents since the active constituents that are responsible for the potent antiproliferative activity have not been much investigated. The seven semipurified fractions were evaluated for antiproliferative activity (Table 3). The HF-2 fraction exhibited potent antiproliferative activity with the IC_50_ value of 6.49 *μ*g/mL which was 2.97-, 4.07-, and 0.08-fold its HF, cisplatin, and doxorubicin, respectively. The HF-4 which gave the strongest metal chelating activity (CC_50_ = 158.45 *μ*g/mL) with the highest phenolic (QE = 169.99 *μ*g/mg) and flavonoid contents (QE = 556.54 *μ*g/mg) was regarded as MA antiproliferative activity (IC_50_ = 59.60 *μ*g/mL). Phenolic and flavonoid compounds might be responsible for the potent antiproliferative activity of* V. denticulata*. The preparative HPLC of HF-2 of* V. denticulata* yielded a single compound, emodin (60.6 mg, *R*_*t*_ 19.62 min) which was characterized by the ^1^H and ^13^C-NMR spectra comparing with the reference data [10, 11]. The chemical structure of emodin was shown in Figure 2. The spectral data of isolated compound was as follow.

Emodin; ^1^H-NMR (400 MHz, DMSO-d_6_): *δ* 2.38 (s, 3H), 6.52 (d, *J* = 1.6 Hz, 1H), 7.05 (s, 1H), 7.11 (s, 1H), 7.43 (s, 1H), ^13^C-NMR (100 MHz, DMSO-d_6_): *δ* 21.96, 108.36, 108.98, 109.74, 113.83, 120.84, 124.53, 133.22, 135.46, 148.51, 161.83, 164.99, 166.84, 181.90, 189.75.

## 4. Discussion

Twenty-three plants were found in high frequency in the anticancer recipes which were selected from the “MANOSROI III” database. Although most recipes were traditionally prepared by boiling with water, in this study these 23 plants were extracted by methanol, a high polarity range solvent under reflux. Then, the wide polarity range of constituents comparing with the water extract will be obtained. The anticancer activities of phytochemicals in some of the 23 plants have been previously reported. Saponins isolated from* A*.* chinensis* stem bark extract had cytotoxic activity on human colon, hepatoma, lung, and gastric cancer cell lines [12]. Brazilin (homoisoflavonoid) from the heartwood of* C. sappan* showed antiproliferative activity against human glioblastoma cells (U87) via apoptosis induction [13]. Thiocolchicoside (colchicinoidal compounds) from the plant* G. superba* exhibited anticancer activity against leukemia, myeloma, squamous cell carcinoma, breast, colon, and kidney cancer cells through inhibition of NF-*κ*B and NF-*κ*B regulated gene products [14]. Rhinacanthin-C (naphthoquinone esters) from the roots of* R. nasutus* gave antiproliferative activity against panel of 10 kinds of cancer cells (KB, Hep-2, MCF-7, HepG2, HeLa SiHa, C-32, LLC, Colon-26, and P388) together with antitumor activity in Meth-A sarcoma bearing mice [15].

As shown in Table 1, the ME of* S*.* collinsae* exhibited the highest antiproliferative activity on HepG2 cell line. According to the NCI criteria, 1 (4.34%) and 8 (34.78%) out of 23 plants were considered as A and MA of antiproliferative activity, respectively, whereas other 14 plant extracts did not exhibit any activity, even though they were the composition of the anticancer recipes. These plants might have activity on other cancers, but not on HepG2 cell line or have other supporting or synergistic effects for cancer treatment, such as antitumor and anti-inflammatory activities or reduced the toxicity of other plants. It has been previously reported that* C*.* fistula* seed extracts have antitumor activity in Ehrlich ascites carcinoma [16], whereas* A. ebracteatus* showed anti-inflammatory activities [17]. The medicinal plants in the recipe N040 such as* C. sappan*,* A. chinensis,* and* Sida rhombifolia* which showed potent antiproliferative activity on HeLa (human cervical adenocarcinoma) cell have been reported to have antiproliferative effect. The antiproliferative activity of the recipe N040 might be due to the bioactive compounds from the mixed medicinal plants in the recipe [18].

After fractionation, all fractions from MEs of the 9 selected plants except* S*.* collinsae* showed higher antiproliferative activity than their corresponding MEs. Although the fraction from* S*.* collinsae* gave lower activity than its ME, its activity was still in the A range. This study indicated that liquid-liquid partition technique appeared to be a suitable purification method to obtain the active anticancer fraction. In Table 2, MF of* G*.* superba* indicated the highest activity. The activity might be mainly due to the colchicine of the plant in the nonpolar fractions [14, 19]. No study on the highly polar fractions of the methanolic extract of this plant has been reported. In this study, not only has the nonpolar fraction (HF) of this plant been investigated, but the higher-polar fraction (BF) which exhibited the high antiproliferative activities on HepG2 cell line was also described.

For antioxidative activities, MF of* C*.* sappan* exhibited the highest DPPH radical scavenging activities. In fact, the similar antioxidative and anticancer activities of* C*.* sappan* have been previously reported [20, 21]. One of the major components of* C*.* sappan* heartwood was homoisoflavanoids [22–24]. HF of* S*.* alata* showed the highest metal chelating activity. The nonpolar constituents such as flavonoids of* S*.* alata* seemed to play a role for the metal chelating activity. Phenolic compounds such as flavonoids, phenolic acids, and tannins are attributed to the antioxidative effect of* S. alata* [25]. ME of* C*.* sappan* and MF of* R*.* nasutus* indicated the highest phenolic and flavonoid contents, respectively. As known, phenolic compounds such as flavonoids, phenolic acids, and tannins are considered as the major contributor to the antioxidative property of vegetables, fruits, or medicinal plants. The antioxidative activity of the phenolic compounds was attributed to its redox properties, which allowed them to act as reducing agents, hydrogen donators, singlet oxygen quenchers, and also metal chelating properties [26]. The strong DPPH radical scavenging and metal chelating activities of ME of* C*.* sappan* and MF of* R. nasutus* might be from the presence of phenolic compounds and flavonoids.

Twelve out of 36 fractions of the 9 selected plants were regarded as A antiproliferative activity (Table 2). The active compounds in some plants which were responsible for the potent antiproliferative activity have been reported. Colchicine from* G. superba*, brazilin and brazilein (homoisoflavonoids) from* C. sappan,* and rhinacanthin-C (naphthoquinone esters) from* R. nasutus* have been reported as active compounds responsible for the antiproliferative activity of their MEs and fractions [13–15, 27]. Besides, stemofoline (stemona alkaloids) from* S. collinsae* has been reported as chemosensitizer agents that increase chemosensitivity in the treatment of multidrug-resistant cancers [28]. However, the antiproliferative constituents in* V*.* denticulata* were not clearly identified, whereas anthraquinones have been found in this plant [29]. This study has been the first description of antiproliferative activity of* V*.* denticulata* on human hepatoma cell line (Hep G2). HF of* V*.* denticulata* which indicated improved activity comparing to its ME was further semipurified. In Table 3, HF-2 fraction exhibited the highest antiproliferative activity with 4.07- and 0.08-fold cisplatin and doxorubicin, respectively, whereas the HF-4 fraction gave the highest metal chelating activity and contained the highest phenolic and flavonoid contents. By preparative HPLC of HF-2 fraction, emodin was isolated as the major constituent. Emodin and its derivatives have been reported as the major constituents of the root bark of* V. denticulata* [30]. The antiproliferative activity on HepG2 cell line of emodin with the GI_50_ of 13.1 *μ*M (3.4 *μ*g/mL) has been indicated [31]. Also, emodin has been shown to have ability to induce the apoptosis on HepG2 and HeLa cell lines [32–34]. Hence, emodin may be responsible for the potent antiproliferative activity on HepG2 cell line. This present study has first described the antiproliferative activity of emodin in Hep G2 cell line. However, the anticancer activity against Hep G2 cell line of emodin isolated from* V. denticulata* in animals has never been reported. Hence, the* in vivo* anticancer activity using Hep G2 cell xenograft nude mice model will be further investigated.

## 5. Conclusion

The antiproliferative activity on Hep G2 cell line of the 23 medicinal plants selected from the “MANOSROI III” database was investigated. Among the 23 MEs,* S*.* collinsae* root extract showed potent with the highest antiproliferative activity in HepG2 cell line at 4.79- and 0.10-fold cisplatin and doxorubicin, respectively. After liquid-liquid partition of the selected 9 MEs, MF of* G*.* superba* gave the highest antiproliferative activity at 50.07- and 1.02-fold cisplatin and doxorubicin, respectively. The 9 selected plants, except* S*.* collinsae,* indicated higher antiproliferative activity than their corresponding MEs. The highest DPPH free radical scavenging (1.09-fold *α*-tocopherol) and metal chelating activities (0.28-fold EDTA) were observed in MF of* C. sappan* and HF of* S. alata*, respectively. After purification of HF of* V*.* denticulata*, emodin was isolated. This compound seemed to be responsible for the antiproliferative activity of* V*.* denticulata*. This study has confirmed not only the traditional use of the Thai/Lanna medicinal plant recipes for cancer treatments, but also the potential of plants selected from these recipes for the further development to modern anticancer drug with antioxidative activities.

## Figures and Tables

**Table 1:** Botanical names, families, part used, yields, and antiproliferative activity on human hepatoma cell line (HepG2) of the 23 methanol extracts of the medicinal plant selected from Thai/Lanna medicinal recipe database “MANOSROI III.”

Plant number	Name of the medicinal plant	Family	Part used	Yield (w/w%)	Voucher specimen number	Local name	English name	Antiproliferative activity on Hep G2				
								IC_50_ (*µ*g/mL)	Folds of		NCI criteria	Rank
									C	D		
1	*Acanthus ebracteatus *Vahl	Acanthaceae	Stem	11.50	MANOSROI#0033	Ngueak Pla Moh	Sea holly	>1000	ND	ND	IA	15
2	*Aegle marmelos *(L.) Correa ex Roxb.	Rutaceae	Fruit	43.88	MANOSROI#0034	Ma Toom	Bael	>1000	ND	ND	IA	15
3	*Albizia chinensis *(Osbeck) Merr.	Leguminosae-*Mimosoideae *	Wood	5.79	MANOSROI#0021	Kang Luang	Silk tree	34.50	0.77	0.02	MA	7
4	*Caesalpinia sappan *L.	Caesalpiniaceae	Wood	14.23	MANOSROI#0022	Nam Khong, Fang	Sappan tree	20.12	1.31	0.03	MA	3
5	*Cassia fistula *L.	Leguminosae	Wood	6.71	MANOSROI#0012	Ratcha Phruek	Golden shower	372.23	0.07	0.00	IA	11
6	*Fibraurea tinctoria *Lour.	Menispermaceae	Wood	7.04	MANOSROI#0035	Kamphaeng Ched Chan	—	89.11	0.30	0.01	MA	9
7	*Gloriosa superba *L.	Colchicaceae	Root	7.92	MANOSROI#0036	Dong Dueng	Flame lily	20.11	1.31	0.03	MA	2
8	*Hydnophytum formicarum *Jack	Rubiaceae	Bulb	17.06	MANOSROI#0037	Hua Roi Ru	Ant plant	129.02	0.20	0.00	IA	10
9	*Nymphoides indica *Ktze.	Gentianaceae	Wood	8.37	MANOSROI#0025	Tab Tao Yai	Water snowflake	>1000	ND	ND	IA	15
10	*Peltophorum pterocarpum *(DC.) Backer ex K. Heyne	Leguminosae-Caesalpinoideae	Wood	10.52	MANOSROI#0023	San Ngoen	Copper pod	395.01	0.07	0.00	IA	13
11	*Polyalthia debilis *Pierre Finet et Gagnep.	Annonaceae	Wood	5.96	MANOSROI#0024	Kluay Tao, Tab Tao	—	>1000	ND	ND	IA	15
12	*Pouzolzia pentandra *Benn.	Urticaceae	Leaf	5.54	MANOSROI#0045	Kob Cha Nang Dang	—	35.51	0.74	0.02	MA	8
13	*Psocarpus tetragonolobus *(L.) DC.	Leguminosae-Papilionoideae	Rhizome	10.07	MANOSROI#0003	Thua Phu	Winged bean	>1000	ND	ND	IA	15
14	*Rhinacanthus nasutus *(L.) Kurz	Acanthaceae	Whole	8.61	MANOSROI#0038	Thong Phan Chang	Snake jasmine	30.91	0.85	0.02	MA	6
15	*Senna alata *(L.) Roxb.	Leguminosae-Caesalpinoideae	Root	21.52	MANOSROI#0030	Chumhet Thet	Candle tree	22.46	1.18	0.02	MA	5
16	*Sida rhombifolia *L.	Malvaceae	Root	7.68	MANOSROI#0020	Ya Khat, Khat Mon	Paddy's lucerne	>1000	ND	ND	IA	15
17	*Smilax glabra *Wall ex Roxb.	Smilaceae	Bulb	17.05	MANOSROI#0039	Khao Yen Tai	China root	>1000	ND	ND	IA	15
18	*Smilax corbularia *Kunth	Smilaceae	Bulb	17.22	MANOSROI#0040	Khao Yen Nuea	Long-leaved greenbrier	>1000	ND	ND	IA	15
19	*Stemona collinsae *Craib	Stemonaceae	Stem	23.00	MANOSROI#0041	Non Tai Yak	—	5.51	4.79	0.10	A	1
20	*Suregada multiflora *(A. Juss.) Baill	Euphorbiaceae	Wood	6.35	MANOSROI#0042	Khan Thong Phayabat	Flase lime	>1000	ND	ND	IA	15
21	*Tiliacora triandra *Diels.	Menispermaceae	Leaf	12.90	MANOSROI#0043	Thao Ya Nang	—	487.85	0.05	0.00	IA	14
22	*Urceola minutiflora *Pierre. (DJ. Middleton)	Apocynaceae	Vine	9.64	MANOSROI#0026	Thao Muak Khao	—	>1000	ND	ND	IA	15
23	*Ventilago denticulata *Wild.	Rhamnaceae	Stem	4.97	MANOSROI#0044	Thaowal Lek	—	20.88	1.26	0.03	MA	4

Standard	Cisplatin (C)							26.41	1	—	—	—
	Doxorubicin (D)							0.54	—	1	—	—

NCI criteria: the criteria indicated 3 types of activity. A: active (IC_50_ < 20 mg/mL), MA: moderately active (IC_50_ = 20~100 mg/mL), and IA: inactive (IC_50_ > 100 mg/mL). ND: not determined. 1 of 23 plants (4.34%) was found active (A) and 8 of 23 plants (34.78%) were found moderately active (MA).

**Table 2:** Antiproliferative activity on HepG2 cell line, DPPH radical scavenging and metal chelating activities, total phenolic and flavonoid contents of 9 methanolic extracts (MEs) and 36 semipurified fractions of the 9 selected medicinal plants selected from Thai/Lanna medicinal plant recipe database “MANOSROI III.”

Plant number	Name of medicinal plant	Hep G2					DPPH				Chelating			Phenolic content		Flavonoid content	
		IC_50_ (*µ*g/mL)	Folds of		NCI criteria	Rank	SC_50_ (*µ*g/mL)	Folds of		Rank	CC_50_ (*µ*g/mL)	Folds of	Rank	QE (*µ*g/mg)	Rank	QE (*µ*g/mg)	Rank
			C	D				T	A			E					
3	*Albizia chinensis* ME	34.50	0.77	0.02	MA	23	59.30	0.40	0.31	8	>500	ND	16	218.51	6	0.81	28
	*A. chinensis* HF	>100	ND	ND	IA	26	>100	ND	ND	12	435.60	0.03	14	12.98	39	ND	31
	*A. chinensis* MF	>100	ND	ND	IA	26	40.20	0.59	0.46	4	>500	ND	16	149.17	9	3.95	19
	*A. chinensis* BF	10.80	2.44	0.05	A	7	47.30	0.50	0.39	6	>500	ND	16	323.63	3	2.13	23
	*A. chinensis* WF	31.50	0.84	0.02	MA	22	87.60	0.27	0.21	11	>500	ND	16	122.73	10	1.13	27

4	*Caesalpinia sappan* ME	20.12	1.31	0.03	MA	14	26.00	0.92	0.70	2	>500	ND	16	475.41	1	22.39	5
	*C. sappan* HF	>100	ND	ND	IA	26	>100	ND	ND	12	>500	ND	16	22.90	31	ND	31
	*C. sappan* MF	27.11	0.97	0.02	MA	20	21.91	1.09	0.84	1	>500	ND	16	61.58	18	9.61	10
	*C. sappan* BF	9.97	2.65	0.05	A	6	26.60	0.89	0.69	3	>500	ND	16	361.69	2	4.62	15
	*C. sappan* WF	>100	ND	ND	IA	26	>100	ND	ND	12	>500	ND	16	64.16	17	0.59	29

6	*Fibraurea tinctoria* ME	89.11	0.30	0.01	MA	25	43.20	0.55	0.42	5	>500	ND	16	305.17	4	2.32	22
	*F. tinctoria* HF	>100	ND	ND	IA	26	>100	ND	ND	12	367.30	0.04	11	16.14	36	ND	31
	*F. tinctoria *MF	19.11	1.38	0.03	A	12	51.10	0.47	0.36	7	>500	ND	16	155.36	8	4.15	17
	*F. tinctoria* BF	21.33	1.24	0.03	MA	17	76.40	0.31	0.24	10	>500	ND	16	260.24	5	4.57	16
	*F. tinctoria* WF	>100	ND	ND	IA	26	62.40	0.38	0.29	9	>500	ND	16	175.41	7	3.91	20

7	*Gloriosa superba* ME	20.11	1.31	0.03	MA	14	>100	ND	ND	12	>500	ND	16	52.83	20	8.32	11
	*G. superba* HF	3.82	6.91	0.14	A	2	>100	ND	ND	12	>500	ND	16	20.00	32	ND	31
	*G. superba* MF	0.53	50.07	1.02	A	1	>100	ND	ND	12	>500	ND	16	59.69	19	3.98	18
	*G. superba* BF	14.73	1.79	0.04	A	9	>100	ND	ND	12	>500	ND	16	36.46	24	ND	31
	*G. superba* WF	>100	ND	ND	IA	26	>100	ND	ND	12	336.60	0.04	8	8.61	41	ND	31

12	*Pouzolzia pentandra* ME	35.51	0.74	0.02	MA	24	>100	ND	ND	12	228.20	0.06	5	5.85	42	5.23	13
	*P. pentandra* HF	18.36	1.44	0.03	A	11	>100	ND	ND	12	78.80	0.16	2	14.41	37	ND	31
	*P. pentandra* MF	11.82	2.23	0.05	A	8	>100	ND	ND	12	>500	ND	16	46.38	22	16.24	7
	*P. pentandra* BF	>100	ND	ND	IA	26	>100	ND	ND	12	>500	ND	16	26.15	28	0.13	30
	*P. pentandra* WF	>100	ND	ND	IA	26	>100	ND	ND	12	>500	ND	16	5.30	44	ND	31

14	*Rhinacanthus nasutus* ME	30.91	0.85	0.02	MA	21	>100	ND	ND	12	264.10	0.05	7	13.72	38	12.76	8
	*R. nasutus* HF	>100	ND	ND	IA	26	>100	ND	ND	12	130.60	0.10	3	17.39	33	ND	31
	*R. nasutus* MF	4.90	5.38	0.11	A	3	>100	ND	ND	12	243.50	0.05	6	35.16	25	35.91	1
	*R. nasutus* BF	>100	ND	ND	IA	26	>100	ND	ND	12	>500	ND	16	17.08	34	ND	31
	*R. nasutus* WF	>100	ND	ND	IA	26	>100	ND	ND	12	398.40	0.03	13	9.24	40	1.85	24

15	*Senna alata* ME	22.46	1.18	0.02	MA	18	>100	ND	ND	12	>500	ND	16	67.68	16	21.84	6
	*S. alata* HF	26.58	0.99	0.02	MA	19	>100	ND	ND	12	45.60	0.28	1	3.63	45	26.19	3
	*S. alata* MF	5.75	4.59	0.09	A	5	>100	ND	ND	12	>500	ND	16	112.97	12	33.73	2
	*S. alata* BF	>100	ND	ND	IA	26	>100	ND	ND	12	362.70	0.04	10	86.89	13	1.40	26
	*S. alata* WF	>100	ND	ND	IA	26	>100	ND	ND	12	186.60	0.07	4	35.09	26	ND	31

19	*Stemona colinsae * ME	5.51	4.79	0.10	A	4	>100	ND	ND	12	391.70	0.03	12	27.45	27	1.69	25
	*S. colinsae* HF	>100	ND	ND	IA	26	>100	ND	ND	12	>500	ND	16	24.08	30	ND	31
	*S. colinsae* MF	14.73	1.79	0.04	A	9	>100	ND	ND	12	>500	ND	16	44.83	23	4.82	14
	*S. colinsae* BF	>100	ND	ND	IA	26	>100	ND	ND	12	>500	ND	16	16.82	35	ND	31
	*S. colinsae* WF	>100	ND	ND	IA	26	>100	ND	ND	12	436.00	0.03	15	5.83	43	ND	31

23	*Ventilago denticulata* ME	20.88	1.26	0.03	MA	16	>100	ND	ND	12	>500	ND	16	83.73	14	10.08	9
	*V. denticulata* HF	19.30	1.37	0.03	A	13	>100	ND	ND	12	>500	ND	16	48.75	21	26.00	4
	*V. denticulata* MF	>100	ND	ND	IA	26	>100	ND	ND	12	>500	ND	16	120.09	11	7.25	12
	*V. denticulata* BF	>100	ND	ND	IA	26	>100	ND	ND	12	>500	ND	16	78.36	15	2.63	21
	*V. denticulata* WF	>100	ND	ND	IA	26	>100	ND	ND	12	343.20	0.04	9	25.14	29	ND	31

Standard	Cisplatin (C)	26.40	1.00	—	—	—	—	—	—	—	—	—	—				
	Doxorubicin (D)	0.54	—	1.00	—	—	—	—	—	—	—	—	—				
	*α*-Tocopherol (T)	—	—	—	—	—	23.80	1.00	0.77	—	NT	ND	—				
	Ascorbic acid (A)	—	—	—	—	—	18.30	1.30	1.00	—	NT	ND	—				
	EDTA (E)	—	—	—	—	—	NT	ND	ND	—	12.90	1.00	—				

Folds of the standard drug were calculated as the ratio between the IC_50_ value of the standard drug and samples. NCI criteria: NCI criteria indicated three types of the activity. A: active (IC_50 _value was less than 20 *µ*g/mL), MA: moderately active (IC_50_ value was between 20 and 100 *µ*g/mL), and IA: inactive (IC_50_ value was more than 100 *µ*g/mL). NT: not tested, ND: not determined.

ME: methanolic extract, HF: *n*-hexane fraction, MF: methanol-water fraction, BF: *n*-butanol fraction, and WF: water fraction.

**Table 3:** Antiproliferative activity on HepG2 cell line, DPPH radical scavenging and metal chelating activities and total phenolic and flavonoid contents of 7 semipurified fractions from the HF of *V*. *denticulata* ME.

Number	Extracts of *Ventilago denticulata *	Hep G2					DPPH				Chelating			Phenolic content		Flavonoid content	
		IC_50_ (*µ*g/mL)	Folds of		NCI criteria	Rank	SC_50_ (*µ*g/mL)	Folds of		Rank	CC_50_ (*µ*g/mL)	Folds of	Rank	QE (*µ*g/mg)	Rank	QE (*µ*g/mg)	Rank
			C	D				T	A			E					
1	ME	20.88	1.26	0.03	MA	3	>100	ND	ND	—	>500	ND	5	83.73	3	10.08	6
2	HF	19.30	1.37	0.03	A	2	>100	ND	ND	—	>500	ND	5	48.75	4	26.00	5
3	HF-1	>100	—	—	IA	5	>100	ND	ND	—	319.60	0.04	3	12.70	7	36.63	2
4	HF-2	6.49	4.07	0.08	A	1	>100	ND	ND	—	>500	ND	5	7.41	8	35.08	3
5	HF-3	>100	—	—	IA	5	>100	ND	ND	—	>500	ND	5	32.75	6	33.52	4
6	HF-4	59.60	0.44	0.01	MA	4	>100	ND	ND	—	158.45	0.08	1	169.99	1	556.54	1
7	HF-5	>100	—	—	IA	5	>100	ND	ND	—	216.40	0.06	2	108.97	2	ND	8
8	HF-6	>100	—	—	IA	5	>100	ND	ND	—	375.33	0.03	4	27.40	5	5.90	7
9	HF-7	>100	—	—	IA	5	>100	ND	ND	—	>500	ND	5	6.54	9	ND	8

Standard	Cisplatin (C)	26.40	1.00	—	—	—	—	—	—	—	—	—	—				
	Doxorubicin (D)	0.54	—	1.00	—	—	—	—	—	—	—	—	—				
	*α*-Tocopherol (T)	—	—	—	—	—	23.80	1.00	0.77	—	NT	ND	—				
	Ascorbic acid (A)	—	—	—	—	—	18.30	1.30	1.00	—	NT	ND	—				
	EDTA (E)	—	—	—	—	—	NT	ND	ND	—	12.90	1.00	—				

Folds of the standard drug were calculated as the ratio between the IC_50_ value of the standard drug and samples. NCI criteria: NCI criteria indicated three types of the activity. A: active (IC_50 _value was less than 20 *µ*g/mL), MA: moderately active (IC_50_ value was between 20 and 100 *µ*g/mL), and IA: inactive (IC_50_ value was more than 100 *µ*g/mL). NT: not tested, ND: not determined.

QE: quercetin equivalent.
